# Radiotherapy with Intensity-Modulated (IMRT) Techniques in the Treatment of Anal Carcinoma (RAINSTORM): A Multicenter Study on Behalf of AIRO (Italian Association of Radiotherapy and Clinical Oncology) Gastrointestinal Study Group

**DOI:** 10.3390/cancers13081902

**Published:** 2021-04-15

**Authors:** Luciana Caravatta, Giovanna Mantello, Francesca Valvo, Pierfrancesco Franco, Lucrezia Gasparini, Consuelo Rosa, Najla Slim, Stefania Manfrida, Francesca De Felice, Marianna A. Gerardi, Stefano Vagge, Marco Krengli, Elisa Palazzari, Maria Falchetto Osti, Alessandra Gonnelli, Gianpiero Catalano, Patrizia Pittoni, Giovani Battista Ivaldi, Alessandra Galardi, Marco Lupattelli, Maria Elena Rosetto, Rita Marina Niespolo, Alessandra Guido, Oreste Durante, Gabriella Macchia, Fernando Munoz, Badr El khouzai, Maria Rosaria Lucido, Annamaria Porreca, Marta Di Nicola, Maria Antonietta Gambacorta, Vittorio Donato, Domenico Genovesi

**Affiliations:** 1 Radiation Oncology Unit, “SS Annunziata” Hospital, “G. D’Annunzio” University of Chieti-Pescara, 66100 Chieti, Italy; luky.gasp@gmail.com (L.G.); consuelo.rosa@unich.it (C.R.); d.genovesi@unich.it (D.G.); 2 Department of Oncology and Radiotherapy, Azienda Ospedaliero Universitaria Ospedali Riuniti, 60002 Ancona, Italy; gio@mobilia.it; 3 Radiotherapy Unit, Clinical Department, CNAO National Center for Oncological Hadrontherapy, 27100 Pavia, Italy; francesca.valvo@cnao.it; 4 Department of Oncology, Radiation Oncology, University of Turin, 10126 Turin, Italy; pierfrancesco.franco@unito.it; 5 Department of Radiotherapy, IRCCS San Raffaele Scientific Institute, 20132 Milan, Italy; slim.najla@hsr.it; 6 “A. Gemelli” IRCCS, UOC di Radioterapia Oncologica, Dipartimento di Diagnostica per Immagini, Radioterapia Oncologica ed Ematologia, Fondazione Policlinico Universitario, 00168 Rome, Italy; stefania.manfrida@policlinicogemelli.it (S.M.); mariaantonietta.gambacorta@policlinicogemelli.it (M.A.G.); 7 Department of Radiotherapy, Policlinico Umberto I, “Sapienza” University of Rome, 00168 Rome, Italy; francesca.defelice@uniroma1.it; 8 Department of Radiotherapy, IEO European Institute of Oncology, IRCCS, 20121 Milan, Italy; marianna.gerardi@ieo.it; 9 Department of Radiation Oncology, IRCCS Ospedale Policlinico San Martino, 16132 Genoa, Italy; stefano.vagge@hsanmartino.it; 10 Division of Radiation Oncology, Department of Translational Medicine, University Hospital “Maggiore della Carità”, University of Piemonte Orientale, 28100 Novara, Italy; marco.krengli@med.uniupo.it; 11 Oncological Referral Center, Radiation Oncology Department, 33081 Aviano, Italy; elisa.palazzari@cro.it; 12 Unit of Radiation Oncology, Sant’Andrea Hospital, Sapienza University of Rome, 00189 Rome, Italy; mattiafalchetto.osti@uniroma1.it; 13 Department of Radiotherapy, Azienda Ospedaliero-Universitaria Pisana, 56124 Pisa, Italy; alessandra.gonnelli@phd.unipi.it; 14 Radiation Oncology Center, IRCCS Multimedica, Sesto San Giovanni, 20099 Milan, Italy; gianpiero.catalano@multimedica.it; 15 Radiation Oncology Unit, Asst Lariana, Ospedale di Como, 22100 Como, Italy; patrizia.pittoni@asst-lariana.it; 16 Radiation Oncology Unit, ICS Maugeri, IRCCS, 27100 Pavia, Italy; giovannibattista.ivaldi@icsmaugeri.it; 17 Department of Radiotherapy, University Hospital, 50134 Florence, Italy; galardia@aou-careggi.toscana.it; 18 Radiation Oncology Section, University of Perugia and Perugia General Hospital, 06156 Perugia, Italy; mlupattelli62@gmail.com; 19 Radiotherapy Unit, Belcolle Hospital, 01100 Viterbo, Italy; mariaelenarosetto@virgilio.it; 20 Radiotherapy Unit, Azienda Ospedaliera San Gerardo, 20900 Monza, Italy; ritamarinaniespolo@gmail.com; 21 Radiation Oncology, IRCCS Azienda Ospedaliero-Universitaria di Bologna, 40138 Bologna, Italy; alessandraguido2008@gmail.com; 22 Azienda Ospedaliera SS, Antonio e Biagio e Cesare Arrigo, 15121 Alessandria, Italy; oreste.durante@ospedale.al.it; 23 Radiation Oncology Unit, Gemelli Molise Hospital—Università Cattolica del Sacro Cuore, 86100 Campobasso, Italy; gabriella.macchia@gemellimolise.it; 24 Department of Radiotherapy, Azienda U. S. L. della Valle d’Aosta, 11100 Aosta, Italy; fmunoz@ausl.vda.it; 25 Radiotherapy and Nuclear Medicine Unit, Veneto Institute of Oncology-IRCCS, 35128 Padova, Italy; badr.elkhouzai@iov.veneto.it; 26 Radiotherapy Unit, Ospedale Sanremo-ASL 1 Imperiese, 18038 Sanremo, Italy; m.lucido@asl1.liguria.it; 27 Laboratory of Biostatistics, Department of Medical, Oral and Biotechnological Sciences, “G. D’Annunzio” University of Chieti-Pescara, 66100 Chieti, Italy; annamaria.porreca@unich.it (A.P.); marta.dinicola@unich.it (M.D.N.); 28 Radiation Oncology, Azienda Ospedaliera San Camillo-Forlanini, 00152 Rome, Italy; vittoriodonato@libero.it; 29 Department of Neuroscience, Imaging and Clinical Sciences, “G. D’Annunzio” University of Chieti-Pescara, 66100 Chieti, Italy

**Keywords:** anal carcinoma, concomitant radio-chemotherapy, intensity-modulated radiotherapy, simultaneous integrated boost

## Abstract

**Simple Summary:**

Concurrent chemo-radiotherapy is the standard treatment in anal cancer. Intensity-modulated radiotherapy (IMRT) was proved to reduce severe, acute and late toxicities. Moreover, IMRT techniques allow for the planning and delivery of a simultaneous integrated boost (SIB), with a differential dose per fraction given to selected sub-regions during the same treatment session. This boost modality provides the chance to employ a dose-painted approach with a reduction in overall treatment time that could result in a potential clinical advantage. Since a large variability in dose prescription to the primary tumor and elective or involved lymph nodes can be found in available guidelines and clinical practice, a multicenter analysis was conducted to evaluate the pattern of care and the impact of radiotherapy parameters on clinical outcomes for anal cancer patients treated with IMRT techniques within a national cohort.

**Abstract:**

A multi-institutional retrospective study was conducted to evaluate the pattern of care and clinical outcomes of anal cancer patients treated with intensity-modulated radiotherapy (IMRT) techniques. In a cohort of 987 patients, the clinical complete response (CR) rate (beyond 6 months) was 90.6%. The 3-year local control (LC) rate was 85.8% (95% CI: 84.4–87.2), and the 3-year colostomy-free survival (CFS) rate was 77.9% (95% CI: 76.1–79.8). Three-year progression-free survival (PFS) and overall survival (OS) rates were 80.2% and 88.1% (95% CI: 78.8–89.4) (95% CI: 78.5–81.9), respectively. Histological grade 3 and nodal involvement were associated with lower CR (*p* = 0.030 and *p* = 0.004, respectively). A statistically significant association was found between advanced stage and nodal involvement, and LC, CFS, PFS, OS and event-free survival (EFS). Overall treatment time (OTT) ≥45 days showed a trend for a lower PFS (*p* = 0.050) and was significantly associated with lower EFS (*p* = 0.030) and histological grade 3 with a lower LC (*p* = 0.025). No statistically significant association was found between total dose, dose/fraction and/or boost modality and clinical outcomes. This analysis reports excellent clinical results and a mild toxicity profile, confirming IMRT techniques as standard of care for the curative treatment of anal cancer patients. Lymph node involvement and histological grade have been confirmed as the most important negative prognostic factors.

## 1. Introduction

The primary aim of curative chemo-radiotherapy (CRT) for stage II-III anal cancer (AC) patients is to achieve locoregional control, while preserving the anal sphincter with intact function and avoiding a colostomy, with a reasonable quality of life. Since definitive CRT showed a high rate of clinical complete response (cCR up 80–90%) and local control (LC = 70–60% at 3 years) in several randomized studies [1,2,3,4,5,6,7,8,9], it currently represents the mainstay of treatment in this clinical setting. However, curative CRT is reported to be associated with a considerable rate of acute toxicities that could require treatment breaks with a prolonged overall treatment time (OTT), potentially affecting clinical outcomes [10,11]. Late effects could also be relevant. Highly conformal radiation modalities, such as intensity-modulated radiation therapy (IMRT), may reduce radiation dose to normal tissue with decreased toxicity and similar locoregional control rates [12,13]. In a large retrospective analysis of 151 patients with AC treated with IMRT, an LC rate of 87% at 3 years was reported with low grade ≥3 acute gastrointestinal (GI) (11%) and skin (20%) toxicities [14]. Moreover, IMRT offers the possibility to deliver a simultaneous integrated boost (SIB), with the chance to give different doses to treatment volumes in the same number of fractions, allowing a safe administration of higher doses to the gross tumor volume (GTV) and with a reduced OTT. Concerning this strategy, the RTOG 0529 trial evaluated dose-painted SIB-IMRT, reporting a significant reduction in ≥G2 acute hematologic, cutaneous and GI toxicity [15]. Several studies have confirmed a benefit in terms of oncological outcomes, toxicity rates and quality of life using IMRT techniques [16,17,18,19,20,21,22]. Based on these results, IMRT planning and delivery represent the standard radiotherapy (RT) option for AC, despite a lack of standardization concerning treatment volumes and doses, especially in the case of an SIB strategy. This variability could probably have an impact on treatment-related toxicity and long-term outcomes. Based on these considerations, a multi-institutional retrospective study was conducted with the aim to evaluate the clinical outcomes, patterns of care and the impact of RT parameters for AC patients treated with different IMRT techniques.

## 2. Results

### 2.1. Patients and Treatment Characteristics

A cohort of 987 consecutive non-metastatic AC patients treated between 2007–2019 was analyzed. Patients were enrolled within 25 different Italian centers. Clinical characteristics for all treated patients are shown in Table 1. 

An Eastern Cooperative Oncology Group (ECOG) performance status (PS) of 0 was reported in 775 patients (78.5%). HIV positivity was identified in 90 (9.1%) of the evaluated patients. In most of the patients, HPV status was not reported (55.5%). Tumors were located within the anal canal in 879 patients (89.1%). Basaloid histology was described in 84 patients (8.5%).

Three hundred thirty patients (33.4%) had cT1-2N0 stage, 106 patients (10.6%) had cT3-4N0 stage and 551 (55.8%) had lymph node involvement. Overall, 633 patients (64.1%) presented with locally advanced disease (LAD) at diagnosis. Twenty-four patients (2.4%) had a single site of clinical abdominal lymph node involvement (lumbar–aortic and/or common iliac lymph nodes).

Imaging modalities used to define tumor (T), nodal (N) and distant metastases (M) stages are shown in Table 2. 

Magnetic resonance imaging (MRI) was used in 541 patients (54.8%) to define T stage; 18FDG-PET was used in 375 patients (38.0%) and 467 patients (47.3%) for N and M stage identification, respectively. 

Treatment characteristics are detailed in Table 3. 

Three hundred and two patients (30.6%) were treated with static or dynamic IMRT, 470 patients (47.6%) with volumetric modulated arc therapy (VMAT) and 215 patients (21.8%) with tomotherapy. A SIB strategy was used in 568 patients (57.5%). An additional sequential boost was administered in 167 patients (16.9%) using external beam RT (EBRT) or brachytherapy (BRT) in 122 patients (12.4%) and 45 patients (4.6%), respectively. Concomitant chemotherapy was administered in 934 patients (94.63%). The mitomycin (MMC) plus 5-fluorouracil (5-FU) or capecitabine regimen was administered in 779 patients (78.92%). 

The total RT dose and daily fractionation prescription were analyzed according to clinical stage at presentation and reported based on the low-, intermediate- and high-risk planning target volumes (PTVs), as defined in the RTOG 0529 study [15]. The median total dose was 55 Gy (range: 45–75). 

In patients with cT1-2 N0 disease, the elective low-risk PTV received a median dose of 45 Gy (range 32.40–48 Gy, 1.40–2 Gy daily) and high-risk PTV received a median dose of 54 Gy (range 37.50–70.40 Gy, 1.80–2.40 Gy daily), with a median total dose of 55 Gy (range 46–70.40 Gy). 

In patients with cT3-T4 N0 disease, the elective low-risk PTV received a median dose of 45 Gy (range 34.50–48 Gy, 1.40–1.80 Gy daily) and high-risk PTV received a median dose of 54 Gy (range 41.40–68.40 Gy, 1.80–2.40 Gy daily), with a median total dose of 55 Gy (range 50–73 Gy). 

In patients with cT1-T2 N+ disease, the elective low-risk PTV received a median dose of 45 Gy (range 35.8–54 Gy, 1.40–2 Gy daily) and high-risk PTV received a median dose of 54 Gy (range 40–68.4 Gy, 1.80–2.40 Gy daily), with a median total dose of 55 Gy (range 45–74.4 Gy).

In patients with cT3-T4 N+ disease, the elective low-risk PTV received a median dose of 45 Gy (range 36–54 Gy, 1.40–2 Gy daily) and high-risk PTV received a median dose of 55 Gy (range 44–72.4 Gy, 1.80–2.40 Gy daily), with a median total dose of 56 Gy (range 45–74.8 Gy).

### 2.2. Treatment Compliance and Toxicity

The median OTT was 45 days (range: 25–115). A treatment interruption of >5 days for toxicity occurred in 186 patients (18.8%). RT was not definitively completed in 67 patients (6.8%). Detailed toxicity profiles are shown in Table 4. 

The most common acute toxicities were grade 2 dermatitis, reported in 491 patients (49.75%), and grade 1 diarrhea, reported in 371 patients (37.6%). Severe (grade > 3) acute toxicity was observed mainly as skin toxicity (desquamation) in 253 patients (25.6%). Other severe toxicities were GI in 65 patients (6.6%) and urogenital in 5 patients (0.5%). Overall, the grade 3–4 acute toxicity rate was 32.7%. The acute hematologic toxicity was reported in 265 patients (26.8%) as grade 1, in 126 patients (12.8%) as grade 2 and in 86 patients (8.7%) as grade >3 toxicity. 

With a median follow-up of 28 months (range 6–138), GI late effects (diarrhea and proctitis) were the most common late toxicities: grade 1 in 205 patients (20.8%), grade 2 in 65 patients (6.6%) and grade 3–4 in 18 patients (1.8%). Grade 1, 2 and 3–4 late skin toxicities were observed in 183 patients (18.5%), 10 patients (1%) and 2 patients (0.2%), respectively. Pelvic bone fractures or density alterations were reported in 16 patients (1.6%). Sexual disorders and dyspareunia occurred in 9 (0.9%) and 3 (0.3%) female patients, respectively. Late anemia and/or thrombocytopenia was described in 5 patients (0.5%).

### 2.3. Treatment Response and Clinical Outcomes

The diagnostic imaging modalities allowing for tumor response evaluation were MRI in 386 patients (39.10%), CT in 184 patients (18.65%), anoscopy in 146 patients (14.80%), endorectal ultrasound (ERUS) in 98 patients (10%) and 18FDG-PET in 71 patients (7.2%). Multiple diagnostic imaging modalities were used in combinations in 90 patients (9.12%).

At 3 months after the start of RT, 536 patients (54.3%) had complete response (CR), 291 patients (29.5%) partial response (PR), 12 patients (1.2%) stable disease (SD), and 13 patients (1.3%) had local progressive disease (PD). For 135 patients (13.7%), data were not available at the 3-month analysis.

At 6 months after the start of RT, 766 patients (77.7%) had CR, 140 patients (14.2%) had PR, 17 patients (1.7%) had SD, and 57 patients (5.8%) had PD. Data were not available at the 6-month analysis for 7 patients (0.7%). The overall clinical CR rate (beyond 6-month evaluations) was 90.6%. 

**Figure 1:**
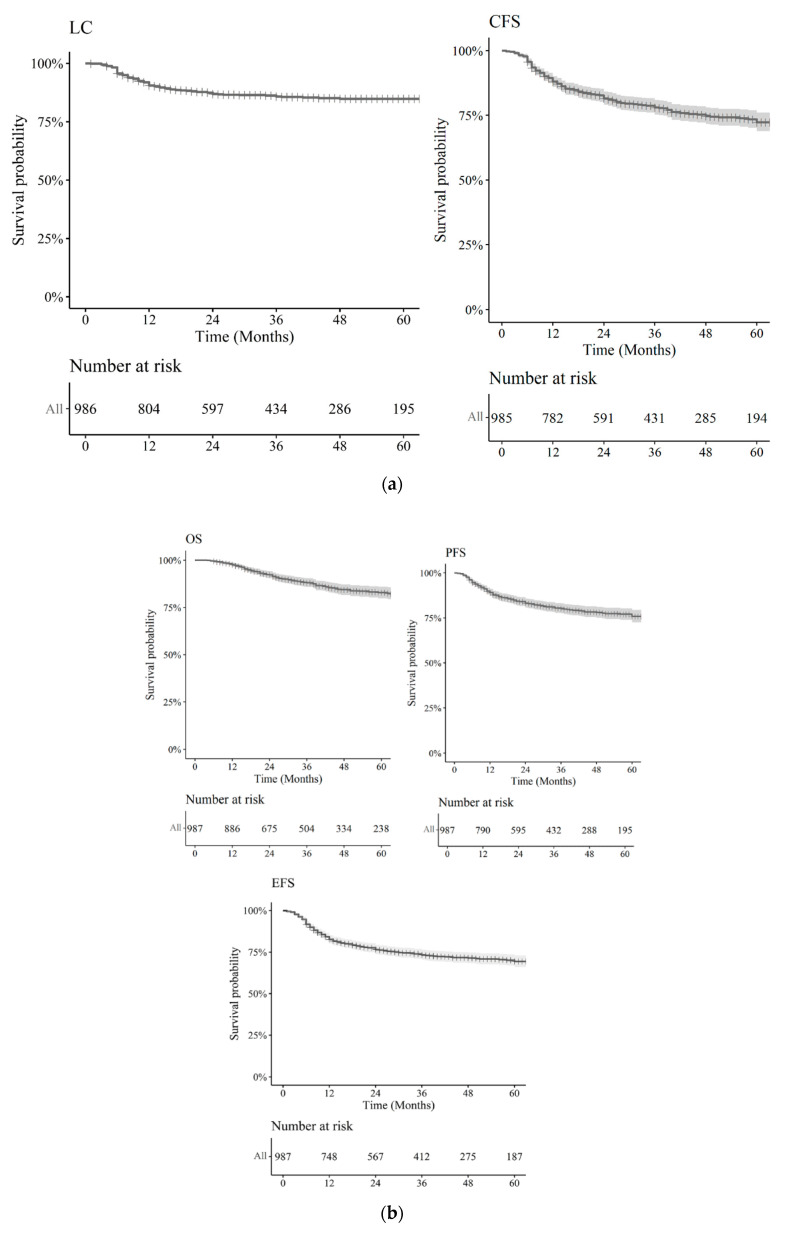
(**a**) Local control (LC), colostomy-free survival (CFS), curves and subjects at risk at 12, 24, 36, 48 and 60 months. (**b**) Overall survival (OS), progression-free survival (PFS), event-free survival (EFS) curves and subjects at risk at 12, 24, 36, 48 and 60 months.

The 2- and 3-year LC rates were 86.9% (95% CI: 85.6–88.2%) and 85.8% (95% CI: 84.4–87.2%), respectively (Figure 1a).

A total of 84 patients underwent a colostomy with abdominoperineal resection in the first year after the start of RT, with an estimated cumulative incidence of colostomy at 12 months of 9.3% (95% CI: 8.2–10.3%). The 2- and 3-year colostomy-free survival (CFS) rates were 81.5% (95% CI: 79.83–83.1%) and 77.9% (95% CI: 76.1–79.8%), respectively (Figure 1a). 

The 2-, 3- and 5-year overall survival (OS) rates were 92.3% (95% CI: 91.3–93.3%), 88.1% (95% CI: 78.8–89.4%) and 82.9% (95% CI: 81.0–84.7%), with 2- and 3-year progression-free survival (PFS) rates of 83.2% (95% CI: 81.6–84.7%) and 80.2% (95% CI: 78.5–82.0%), respectively (Figure 1b). Overall, the 2-, 3- and 5-year event-free survival (EFS) rates were 76.6% (95% CI: 73.8–79.4%), 73.4% (95% CI: 70.5–76.5%) and 69.4% (95% CI: 66.0–73.0%), respectively.

### 2.4. Univariate and Multivariate Analyses

As study variables, we evaluated age, gender, ECOG PS, HPV and HIV status, clinical stage (T1-T2 vs. T3-T4), lymph node involvement (N0 vs. N+), histological grade (G1-G2 vs. G3), and treatment parameters, such as total dose and treatment duration (OTT: <45 vs. ≥45 days). 

Univariate analysis assessing overall clinical CR (after 6 months) showed that histological grade 3 was associated with a low probability of CR (odds ratio (OR) 0.11, 95% CI 0.03–0.52, *p* = 0.030). In addition, node-positive patients had a significantly lower probability of CR than patients with uninvolved lymph nodes (OR 0.45, 95% CI 0.25–0.76, *p* = 0.004). Finally, a significant association with disease extension was reported (OR 0.10, 95% CI 0.04–0.27, *p* < 0.001 and OR = 0.50, 95% CI 0.28–0.85, *p* = 0.013 for extended disease (ED) and LAD, respectively). 

**Figure 2:**
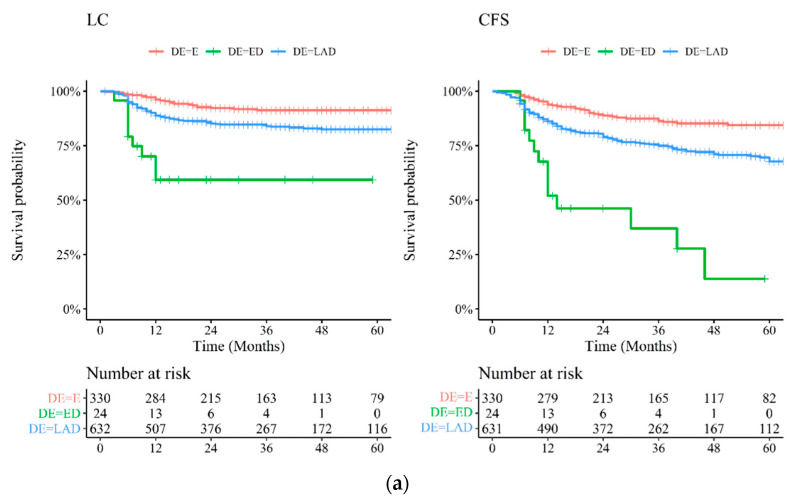

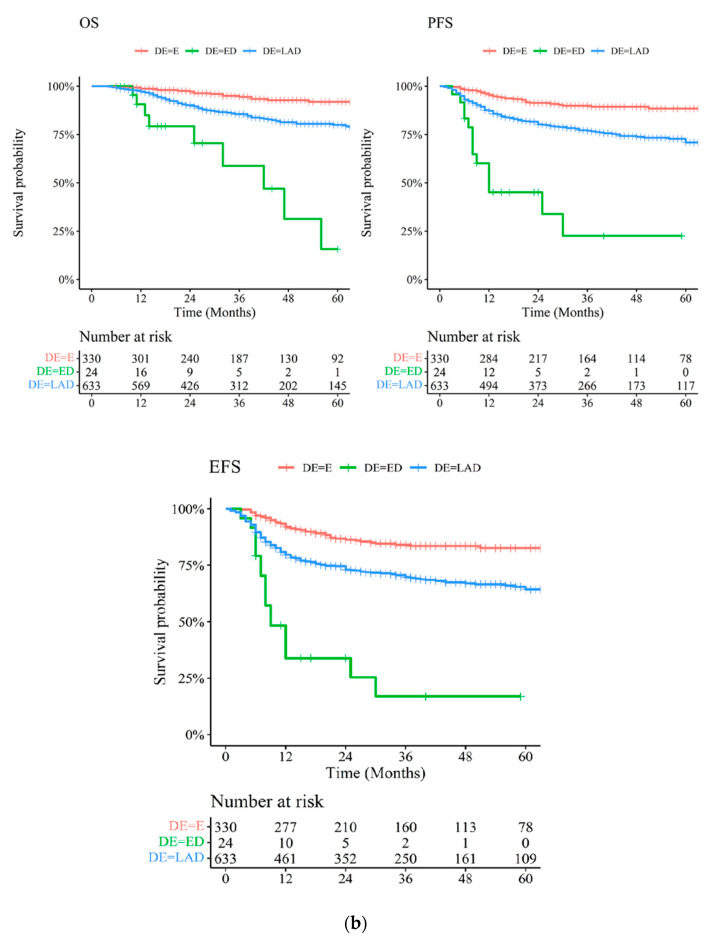
(**a**) Kaplan–Meier survival curves stratified by disease extension and risk table at 12, 24, 36, 48 and 60 months for local control (LC) and colostomy-free survival (CFS). Early disease: T1-T2 tumors; LAD = locally advanced disease (T3-T4 or N+ = node-positive tumors); ED = extended disease (lumbo–aortic and/or common iliac lymph nodes). (**b**) Kaplan–Meier survival curves stratified by disease extension and risk table at 12, 24, 36, 48 and 60 months for overall survival (OS), progression-free survival (PFS) and event-free survival (EFS). Early disease: T1-T2 tumors; LAD = locally advanced disease (T3-T4 or N+ = node-positive tumors); ED = extended disease (lumbo–aortic and/or common iliac lymph nodes).

As shown in Table 5 on univariate analysis, a statistically significant lower CFS, OS, PFS and EFS was found for patients with poor ECOG PS and an advanced stage. Lymph node involvement (regardless of T-stage) and histological grade 3 were associated with a lower LC rate (*p* < 0.001 and *p* = 0.025, respectively). Age >68.5 (cut-off set at 68.5 years as median age of population study) showed a trend for a lower PFS (*p* = 0.052). Moreover, the correlation between clinical outcomes and disease extension is shown in Figure 2a,b.

No statistically significant association was found between RT total dose and/or boost modality (SIB) and clinical outcomes. OTT > 45 days showed a trend with a lower PFS (*p* = 0.050) and was significantly associated with a lower EFS (*p* = 0.030) (Table 6).

According to the multivariate analysis, lymph node involvement negatively affected all clinical outcome measures (LC, CFS, OS, PFS and EFS). Age > 68.5 (cut-off set at 68.5 years as median age of population study) and pathological grade 3 were confirmed as negative prognostic factors for PFS (*p* = 0.019) and LC (*p* = 0.032), respectively.

Investigating the relationships between toxicity (all grades) and the clinical and dosimetric parameters due to the high dimensionality of the comparisons, we calculated ORs and CIs (95%) considering a significant *p*-value of *p* < 0.001. In particular, disease extension results were associated with acute skin toxicity (ED vs. Early: OR = 4.84, 95% CI 2.13–12.04; LAD vs. Early: OR = 0.60, 95% CI 0.46–0. 76), acute gastrointestinal toxicity (LAD vs. Early: OR = 0.51, 95% CI 0.4–0.65) and with acute urological toxicity (LAD vs. Early: OR = 0.36, 95% CI 0.27–0.46). Disease extension results were also associated with skin (ED vs Early: OR = 19.64, 95% CI 8.3–48.63) and subcutaneous late toxicity (LAD vs. Early: OR = 0.43, 95% CI 0.3–0.61).

## 3. Discussion

CRT provided high rates of complete responders (up to 80–90%) in several trials [1,2,3,4,5,6,7,8,9] and currently represents the standard treatment for stage II–III AC patients. Radiotherapy delivery in anal cancer is complex because of the target shape and the proximity to dose-sensitive organs at risk (OARs), such as bowel, femoral heads, bladder, genitalia and perineal skin. IMRT techniques, modulating the beam fluence during delivery, offer the possibility of a dose-painted treatment with high doses to the tumor, minimizing the dose to surrounding OARs. This potentially allows the reduction of acute and late adverse events, improving the therapeutic ratio. The consequent decrease in acute toxicity improves treatment compliance, with a decrease in interruptions and a shorter OTT, with a potential beneficial effect on clinical outcomes. Several studies have shown promising results using this approach [12,13,14,15,16,17,18,19,20,21,22], and it should be considered the standard of care for anal cancer [23].

In our study, all patients were treated with IMRT techniques. 

We observed a substantial clinical response rate: 6-moCR of 77.7% and an overall clinical CR rate (beyond 6-month evaluations) of 90.6%, in agreement with previous studies. 

The ACT II trial reported a 26-week CR rate of 89.6% in the cisplatin group and 90.5% in the MMC group, showing that patients with cCR at 26 weeks had a superior 5-year OS compared to patients without cCR [7]. Based on these data, the European Society for Medical Oncology (ESMO) guidelines recommend a clinical evaluation for CR and endorsed a watchful wait approach, stating that partial regression can be managed by a close follow-up to confirm that complete regression occurs within 6 months [23].

Five-year OS in patients with stage II-III anal squamous cell cancer, treated with CRT, is approximately 75%. RTOG 9811 and ACT II used conformal RT techniques with concurrent 5-FU and MMC and reported 5-year OS rates of 78.3% and 79.0%, respectively [6,8,9]. Long term results (median follow-up of 49 months) of a single institution study of 99 patients, treated with dose-painted IMRT according to the RTOG 0529 trial, showed a 4-year OS of 85.8% [17]. Moreover, in an Italian mono-institutional cohort of 87 patients treated with SIB-IMRT, the 3-year OS rate was 79%, with a 3-year CFS and LC rate of 64% and 69%, respectively [18]. In our study, the estimated 3- and 5-year OS rates were 88.09% (95% CI: 78.76–89.42) and 82.86% (95% CI: 81–84.72), respectively. Consistent results in terms of 3-year LC (85.84%; 95% CI: 84.43–87.25), 3-year CFS (77.94%; 95% CI: 76.07–79.81) and 3-year PFS (80.24; 95% CI: 78.5–81.98) rates were also observed.

Several trials reported that high conformal techniques, such as IMRT, VMAT and tomotherapy, require an accurate definition and delineation of treatment volumes to maintain or potentially improve locoregional control. In AC patients, the primary tumor site, pelvic and inguinal nodes are the main areas of recurrence and could represent three different targets for RT planning. Recommendations based on expert views concerning the primary tumor and elective lymph nodes delineation have been provided [24,25]. On the other hand, no consensus is available from randomized trials regarding the optimal total dose and daily fractionation in the different disease stages, although early-stage disease often receives lower doses, while larger and more advanced tumors are treated with higher doses [1,2,3,4,5,6,7,8].

Data from randomized trials showed that CRT with relatively low total radiation doses (30–50 Gy) and the addition of MMC to 5FU resulted in high LC rates in small tumors (<4 cm) [1,2]. An LC rate ranging between 45–55% has been reported with a modest dose of 45 Gy in 25 fractions and the boost dose modulated according to treatment response [1,2,3,4]. In the RTOG 8704, 9811 phase III trials (median RT dose of 48 Gy, without planned gap) in patients with T3, T4, node-positive disease and residual tumor, an additional boost of 9 to 14 Gy (2.0 Gy/daily, total dose of 54 to 59 Gy) was delivered to the primary tumor/nodal mass [4,6]. Prophylactic irradiation of inguinal lymph nodes up to a dose of 45 Gy increased LC and is recommended, but it remains unclear whether boosting the radiation dose to >50 Gy in patients with good response will improve the results [4,6]. Higher doses above 56 Gy may provide better local control, but they could be associated with an increased toxicity [26], and doses >59 Gy were not shown to provide additional benefits [27].

In our study, patients were treated with a median total dose of 55 Gy (range: 45–75). SIB was delivered in 568 patients (57.5 %), and an additional sequential EBRT or BRT boost was administered in 167 patients (16.9%). Concomitant chemotherapy was administered in all patients with MMC plus 5-FU or the capecitabine regimen in 779 patients (78.92%). 

The treatment was well tolerated, reporting grade 3 acute skin, GI and urogenital toxicity in 251 patients (25.4%), 61 patients (6.2%) and 5 patients (0.5%), respectively. In RTOG 0529, aiming to evaluate the tolerability of a dose-painted IMRT treatment, acute ≥G3 skin and gastrointestinal toxicity rates were 23% and 21%, respectively [15]. Overall, our analysis showed grade 3–4 acute toxicity in 32.7% of patients, related mainly to local skin toxicity (25.6%). Grade 3–4 acute hematologic toxicity in our study was also limited (8.7%), despite the fact that selective approaches to spare the bone marrow were not routinely used in some institutions [28].

The good toxicity profile seems noteworthy considering the median OTT of 45 days (range: 25–115), with a treatment interruption of >5 days for toxicity in 186 patients (18.8%). Nine hundred twenty patients (93.2%) completed the planned RT treatment with a high compliance to treatment. These data confirmed the treatment tolerability with a low rate of interruptions with IMRT techniques, even when SIB is delivered with consequent reduced OTT. 

The SIB strategy, allowing for both gross tumor volume and elective volumes to receive different total doses in the same number of fractions, could require that different elective nodal volumes are treated with a varying dose per fraction [14,15]. Historically, anal cancer has been treated with doses of 1.8 Gy per fraction, using a shrinking field technique over the course of treatment. Using IMRT with SIB to treat different target volumes with different daily doses often results in some areas receiving less than conventional fractional doses of radiation (i.e., <1.80 Gy). In the phase II RTOG 0529 study [15], some patients received, to some elective nodal volumes, a fractional dose of 1.5 Gy without loss of effectiveness in LC and disease-free survival rates compared to RTOG 9811 [6]. The median OTT in the study was 43 days compared with 49 days in the RTOG 9811.

In AC patients, the reduction of OTT could lead to a clinical benefit, whereas a longer OTT could be detrimental to the likelihood of receiving a colostomy and LC [10,11] due to an accelerated repopulation that could occur after irradiation and may lead to a loss in tumor control [29]. Data pooling from the RTOG-8704 and the RTOG-9811 studies showed that OTT was positively associated with time to colostomy failure but not with OS or CFS [10]. Although our analysis did not show a significant correlation between OTT prolongation and CR rate, a detrimental trend effect was observed between OTT > 45 days and PFS (*p* = 0.050) and was significantly associated with a lower EFS (*p* = 0.030), confirming the benefit of “gap” avoidance techniques, such as IMRT-SIB [11].

Recently, according to the UK IMRT guidance [30], a consecutive cohort of 385 patients was retrospectively analyzed. All treatments were delivered by SIB in 28 fractions, with T1-2 N0 receiving 50.4 Gy to the gross primary tumor and T1-2 N+ or T3-4 N-any receiving 53.2 Gy. Involved nodes received 50.4 Gy if 3 cm large; uninvolved pelvic nodes (PTV-elective), including mesorectal, obturator, external and internal iliac, inguinal and presacral, received 40 Gy since March 2014 (dose per fraction: 1.4 Gy). As reported by the authors, this was a biologically equivalent dose to 30.6 Gy in 17 fractions delivered in the ACT2 trial, using an alpha–beta ratio of 8 and a loss of 0.7 Gy per day after 20 fractions. Only two isolated lymph node relapses have been reported with this low dose per fraction approach on elective volume [31]; then, the efficacy of doses less than 1.80 Gy per day are assumed.

Parallel to these studies, in our cohort the elective low-risk PTV received less than 1.80 Gy per day in more than half of the patients. No statistically significant correlation was found between boost modality (SIB), total RT dose (grouping patients receiving less than, equal to and more than 54 and 55 Gy) or dose per fraction (grouping patients receiving hazard ratio (HR) PTV less than, equal to and more than 2 Gy/fraction, and LR PTV less than, equal to and more than 1.8–2 Gy/fraction) and clinical response and outcomes.

Even though a certain heterogeneity in the dose volume prescription of elective PTVs has been reported in our study, this factor did not affect treatment response, and the several RT schedules used seem to be equivalent in terms of clinical outcomes. Based on these results, although recommendations about optimal total dose and fractionation cannot be provided, our study confirmed that low fractional doses may be appropriate for clinically negative areas when using IMRT for AC with concurrent chemotherapy. The ongoing PLATO integrated protocol looking at dose escalation in LAD and dose de-escalation in early small-node-negative disease is currently set up in the UK, and in the future will inform dose fraction optimization for AC. Finally, we are currently performing a national survey to explore the gray areas in the pattern of care of AC and to define a clinical practice consensus, especially in terms of treatment doses and volumes.

Data on the HPV status of more than half of our patients were missing, which is probably related to the retrospective nature of the study, and this could explain the positive/negative HPV-rate in our analysis. Indeed, AC is reported to be associated with HPV infection in 70–90% of cases, with HPV16 as the most common sub-type. This could also have had an impact on the non-statistically significant correlation with clinical outcomes in the two subgroups.

Histological grade 3 and lymph node involvement (with any T) were associated with a lower probability of CR (OR 0.11, 95% CI 0.03–0.52, *p* = 0.030 and OR 0.45, 95% CI 0.25–0.76, *p* = 0.004, respectively) and lower LC (*p* < 0.001 and *p* = 0.025, respectively). Lymph node involvement remained negatively associated with all clinical outcomes (LC, CFS, OS, PFS and EFS) also in the multivariate analysis. Advanced stage was confirmed to be a negative prognostic factor for CFS, OS, PFS and EFS. Furthermore, as reported in other studies, nodal involvement and advanced stage results were significant predictors for LC, OS and PFS [32]. Moreover, disease extension results, probably due to large volumes treated at high doses, were associated with higher risk of toxicity.

## 4. Materials and Methods 

The study was designed as a retrospective research project named “RAINSTORM: RAdiotherapy with INtenSiTy mOdulated (IMRT) techniques in the treatment of anal caRcinoMa: a multicenter retrospective observation study” approved by the Ethical Committee of the “SS Annunziata” Hospital, “G. D’Annunzio” University, Chieti, Italy, and all joined centers.

All patients had histologically confirmed AC (both anal canal and margin). Pretreatment data, including age, gender, performance status as per Eastern Cooperative Oncology Group (ECOG), histological type, human papilloma virus (HPV) and human immunodeficiency virus (HIV) status, were collected. For diagnosis and staging, computed tomography scan (CT), endorectal ultrasound (ERUS), magnetic resonance imaging (MRI) and 18F-fluorodeoxyglucose positron emission tomography (18FDG-PET) were employed. Patients were staged according to the UICC TNM staging system 6th edition 2002 and 7th edition 2009 and classified in early disease (E: T1-2N0) and locally advanced disease (LAD: T3-4 or N+). Patients with T1N0 tumors of the anal margin were excluded in case they received local excision. Patients with a single site of clinical abdominal lymph node involvement (lumbar–aortic and/or common iliac lymph nodes) were included and classified in extended disease (ED). 

### 4.1. Treatment Characteristics

The total radiotherapy dose and daily fractionation prescription were investigated according to the planning target volumes (PTVs) as defined in the RTOG 0529 study [15] (low-, intermediate- and high-risk volumes) and analyzed based on clinical stage at presentation. All patients were treated with one of the IMRT modalities available in their center: static or dynamic IMRT, volumetric modulated arc therapy (VMAT) or helical tomotherapy (HT). Timing and modalities of further boost were also investigated. Detailed data concerning concomitant chemotherapy were collected. 

### 4.2. Statistical Analysis

The primary endpoints were the clinical complete response rate at 6 months (6-moCR) and colostomy-free survival (CFS). CFS was defined as the time between the start of CRT and the date of colostomy, death or last follow-up in which the patient was known to be colostomy-free. Secondary endpoints were overall survival (OS) and acute and late toxicity. OS was calculated in all patients from the date of diagnosis to the date of death from any cause or the last follow-up; toxicities were retrospectively graded according to Common Terminology Criteria for Adverse Events (CTCAE) Version 4.0 by the treating physicians [33]. Acute toxicities were defined as occurring within 90 days of treatment and late toxicities as occurring after 90 days.

Tumor response assessment was investigated at 3 and 6 months after the start of CRT and evaluated with the response evaluation criteria in solid tumors (RECIST) v1.1 [34]. LC was defined as the time between the start of CRT and the date of the first documented tumor recurrence or persistent local disease after CRT. Progression-free survival (PFS) was defined as all progressive diseases, local recurrence, metastases, or death from any cause [35,36]. Event-free survival (EFS) was then calculated for all documented events (local recurrence, metastases, colostomy or death). For patients in which no event occurred, we defined the follow-up time interval as the time elapsed until the last scheduled follow-up visit.

The univariate ordinal logistic regressions and binomial logistic regressions were used to study the relationships between toxicities and the main variables of interest. The results of models were expressed as an odds ratio (OR) and a relative 95% confidence interval (95% CI). Due to the high dimensionality of the comparisons, we considered a *p*-value < 0.001.

Univariate logistic regression models were applied to determine the study variables predictive of the clinical complete response rate at 6 months (6-moCR). The results of models were expressed as an odds ratio (OR) and a relative 95% confidence interval (95% CI). The Kaplan–Meier method was used to calculate the rates of LC, CFS, OS, PFS and EFS at different time points. Univariate and multivariate analyses were performed using the Cox proportional hazards model to determine independent prognostic factors that had a significant impact on clinical outcomes. Calculating the exponential of the regression coefficients from the Cox model provided an estimate of the hazard ratio (HR) and the 95% confidence interval (95% CI). Multicollinearity between variables was also tested using the variation inflation factor (VIF). The stability of the models was guaranteed by backward fitting procedure. All statistical analyses were performed using R statistical software (version 3.1.2.; R Foundation for Statistical Computing, Vienna, Austria). All *p*-values were two-tailed and a *p*-value < 0.05 was considered indicative of a statistically significant association.

## 5. Conclusions

Related to its retrospective design, our study has some limitations, such as RT dose heterogeneity and boost delivery modality reporting. Moreover, given the variable follow-up, there may have been difficulty in analyzing toxicity rates, particularly in reporting acute hematological toxicity, compliance to chemotherapy and overall late toxicities.

On the other hand, to our knowledge, this analysis reports the largest cohort of patients treated with IMRT techniques showing an excellent clinical result and a good toxicity profile, confirming IMRT techniques as standard of care for curative treatment of anal cancer patients. Disease extension results associated with higher risk of toxicity and lymph node involvement and high histological grade have been confirmed as the most impactful negative prognostic factors for complete clinical response in LC, CFS, OS, PFS and EFS.

## Figures and Tables

**Table 1:** Patient and tumor characteristics.

Patient and Tumor Characteristics		*n* = 987	%
Age = median 68.5 years (range: 54.00–80.00)			
Gender	Male	281	28.4
	Female	706	71.5
ECOG Performance Status	0	775	78.5
	1	197	19.9
	2	15	1.5
HPV	Negative	210	21.3
	Positive	229	23.2
	NR	548	55.5
HIV	Negative	747	75.7
	Positive	90	9.1
	NR	150	15.2
Tumor site	Anal Canal	879	89.1
	Anal Margin	108	10.9
Histology	Squamous	880	89.1
	Basalioid	84	8.5
	Other	23	2.3
Grading	G1	68	6.9
	G2	328	33.2
	G3	233	23.6
	NR	358	36.3
TNM Stage	T1-T2, N0	330	33.4
	T3-T4, N0	106	10.7
	Any T, N+	551	55.8
Disease extension	Early stage	330	33.4
	LAD	633	64.1
	ED	24	2.4

Legend: ECOG = Eastern Cooperative Oncology Group; HPV = human papilloma virus; HIV = human immunodeficiency virus; NR = non-reported; early disease: T1-T2 tumors; LAD = locally advanced disease (T3-T4 or N+ = node-positive tumors); ED = extended disease (lumbar–aortic and/or common iliac lymph nodes involvement).

**Table 2:** Imaging modalities used to define tumor (T), nodal (N) and distant metastases (M) stages.

Staging		N = 987 (%)		
		T	N	M
**Single Imaging modality**	CT	143 (14.5)	186 (18.8)	402 (40.7)
	MRI	541 (54.818)	321 (32.5)	12 (1.2)
	ERUS	126 (12.8)	24 (2.4)	14 (1.4)
	18FDG-PET	99 (10.0)	375 (38)	467 (47.3)
Multiple Imaging modalities *		78 (7.9)	79 (8.2)	81 (8.2)
**Missing data**		0 (0.0)	2 (0.02)	11 (1.1)

Legend: CT = computed tomography scan; MRI = magnetic resonance imaging; ERUS = endorectal ultrasound; 18F-fluorodeoxyglucose positron emission tomography (18FDG-PET); *: all used diagnostic imaging in combinations.

**Table 3:** Treatment details.

Treatment Details		*n* = 987	%
Median Total Dose 55 Gy (range: 45–75)			
IMRT modalities	static or dynamic IMRT	302	30.6
	VMAT	470	47.6
	HT	215	21.8
Boost modalities	Sequential	252	25.5
	SIB	568	57.5
	SIB+ Sequential	122	12.4
	SIB+ Sequential BRT	45	4.6
Concomitant chemotherapy	MMC + 5-FU	634	64.2
	MMC + Capecitabine	145	14.7
	CDDP + 5-FU	21	2.1
	CDDP + Capecitabine	67	6.8
	MMC	2	0.2
	5-FU	8	0.8
	CDDP	3	0.3
	Capecitabine	29	2.9
	Other	21	2.1
	NR	4	0.4
	None	53	5.4

Legend: IMRT = intensity modulated radiation therapy; VMAT = volumetric modulated arc therapy; HT = helical tomotherapy; SIB = simultaneous integrated boost; MMC = mitomycin C; 5-FU = 5-fluorouracil; CDDP = cisplatin; RT = radiotherapy; NR = not reported.

**Table 4:** Acute and late toxicities retrospectively graded according to Common Terminology Criteria for Adverse Events Version 4.0 (*n* = 987).

*n* = 987 (%)						
Grade	G0	G1	G2	G3	G4	G3 and G4
ACUTE TOXICITY						
Skin	107 (10.8)	136 (13.8)	491 (49.7)	251 (25.4)	2 (0.2)	253 (25.6)
Gastrointestinal	232 (23.5)	371 (37.6)	319 (32.3)	61 (6.2)	4 (0.4)	65 (6.6)
Urogenital	552 (55.9)	334 (3.8)	96 (9.7)	5 (0.5)	0 (0.00)	5 (0.50)
Hematologic	510 (51.7)	265 (26.8)	126 (12.8)	70 (7.1)	16 (1.6)	86 (8.7)
LATE TOXICITY						
Skin	792 (80.2)	183 (18.5)	10 (1.0)	2 (0.2)	0 (0.0)	2 (0.2)
Subcutaneous tissue	841 (85.2)	130 (13.2)	13 (1.3)	2 (0.2)	1 (0.1)	3 (0.3)
Gastrointestinal	699 (70.8)	205 (20.8)	65 (6.6)	17 (1.7)	1 (0.1)	18 (1.8)
Urogenital	905 (91.7)	65 (6.6)	13 (1.3)	3 (0.3)	1 (0.1)	4 (0.4)

**Table 5:** Univariate analysis between patient characteristics and clinical outcomes.

Variable	LC			CFS			OS			PFS			EFS		
	HR	95% CI	*p*-Value	HR	95% CI	*p*-Value	HR	95% CI	*p*-Value	HR	95% CI	*p*-Value	HR	95% CI	*p*-Value
Gender (ref. Male)															
Female	0.77	(0.54–1.09)	0.160	0.73	(0.54–0.96)	**0.026**	0.65	(0.45–0.94)	**0.022**	0.74	(0.54–0.98)	**0.050**	0.75	(0.58–0.97)	**0.031**
Age (ref. < 68.5 year)															
≥68.5 year	0.88	(0.62–1.25)	0.491	1.07	(0.82–1.40)	0.604	1.33	(0.93–1.89)	0.111	1.33	(1.00–1.78)	**0.050**	1.15	(0.90–1.47)	0.276
ECOG PS (ref. 0)															
1	1.45	(0.96–2.16)	0.071	1.43	(1.04–1.96)	**0.025**	1.59	(1.06–2.38)	**0.025**	1.55	(1.12–2.16)	**0.009**	1.47	(1.10–1.95)	**0.008**
2	1.60	(0.39 2.16)	0.511	2.50	(1.02–6.09)	**0.044**	4.23	(1.55–11.54)	**0.005**	2.31	(0.85–6.26)	0.098	2.02	(0.83–4.91)	0.121
HIV (ref. no)															
Yes	1.29	(0.73–2.26)	0.381	1.39	(0.92–2.08)	0.117	2.06	(1.28–3.28)	**0.003**	1.67	(1.10–2.52)	**0.015**	1.35	(0.93–1.98)	0.118
HPV (ref. No)															
Yes	1.11	(0.66–1.87)	0.689	1.01	(0.65–1.54)	0.976	1.34	(0.74–2.42)	0.327	0.91	(0.57–1.43)	0.671	0.90	(0.61–1.32)	0.595
Histology (ref. Squamous)															
Basaloid	0.66	(0.32–1.35)	0.255	0.73	(0.43–1.23)	0.242	0.74	(0.37–1.45)	0.379	0.75	(0.43–1.32)	0.320	0.76	(0.47–1.22)	0.256
Histological Grade (ref. 1)															
2	2.60	(0.80–8.42)	0.111	0.97	(0.55–1.68)	0.904	1.03	(0.50–2.10)	0.941	1.07	(0.57–1.98)	0.837	1.07	(0.63–1.83)	0.800
3	3.83	(1.18–12.42)	**0.025**	0.97	(0.54–1.72)	0.917	1.14	(0.55–2.39)	0.720	1.32	(0.70–2.47)	0.391	1.17	(0.67–2.02)	0.582
Stage (ref. cT1-2N0)															
cT3-4N0	1.76	(0.90–3.44)	0.098	2.39	(1.49–3.82)	**<0.001**	3.44	(1.84–6.39)	**<0.001**	2.69	(1.59–4.54)	**<0.001**	2.43	(1.57–3.76)	**<0.001**
Any T N+	2.37	(1.52–3.68)	**<0.001**	2.26	(1.61–3.18)	**<0.001**	2.90	(1.78–4.72)	**<0.001**	2.76	(1.88–4.07)	**<0.001**	2.36	(1.72–3.24)	**<0.001**
Disease extension (ref. Early)															
ED	7.59	(3.53–16.31)	**<0.001**	7.90	(4.30–14.50)	**<0.001**	12.50	(5.66–27.60)	**<0.001**	11.27	(5.98–21.22)	**<0.001**	8.44	(4.85–14.69)	**<0.001**
LAD	2.12	(1.36–3.29)	**0.001**	2.14	(1.52–2.99)	**<0.001**	2.78	(1.71–4.50)	**<0.001**	2.55	(1.74–3.74)	**<0.001**	2.22	(1.62–3.03)	**<0.001**

Legend: ECOG = Eastern Cooperative Oncology Group; HPV = human papilloma virus; HIV = human immunodeficiency virus; early disease: T1-T2 tumors; LAD = locally advanced disease (T3-T4 or N+ = node-positive tumors); ED = extended disease (lumbo–aortic and/or common iliac lymph nodes). HR = hazard risk; 95% CI = 95% confidence interval. In bold, statistically significant values (*p* < 0.05).

**Table 6:** Univariate analysis treatment characteristics and clinical outcomes.

Variable	LC			CFS			OS			PFS			EFS		
	HR	95% CI	*p*-Value	HR	95% CI	*p*-Value	HR	95% CI	*p*-Value	HR	95% CI	*p*-Value	HR	95% CI	*p*-Value
OTT (Ref. <45)															
≥45	1.13	(0.80–1.61)	0.478	1.22	(0.93–1.60)	0.140	1.23	(0.86–1.75)	0.243	1.33	(1.00–1.77)	**0.050**	1.31	(1.03–1.68)	**0.030**
Total dose 54 Gy (ref. ≤ 54 Gy)															
>54 Gy	0.81	(0.57–1.15)	0.238	0.90	(0.69–1.18)	0.463	0.85	(0.60–1.22)	0.383	1.03	(0.77–1.39)	0.821	0.98	(0.76–1.26)	0.882
Total dose 55 Gy (ref. ≤ 55 Gy)															
>55 Gy	0.81	(0.57–1.15)	0.238	0.90	(0.69–1.18)	0.463	0.85	(0.60–1.22)	0.383	1.03	(0.77–1.39)	0.821	1.02	(0.79–1.30)	0.904
Dose/Fraction HR PTV (ref. 1.8–2 Gy)															
>2 Gy)	0.79	(0.56–1.12)	0.187	0.89	(0.67–1.17)	0.385	0.88	(0.61–1.25)	0.471	0,93	(0.69–1.25)	0.647	0.91	(0.71–1.17)	0.452
Dose/Fraction LR PTV (ref. 1.8–2 Gy)															
<1.8 Gy	0.96	(0.67–1.39)	0.835	0.98	(0.74–1.31)	0.908	0.74	(0.52–1.06)	0.102	0.78	(0.58–1.04)	0.090	1.01	(0.67–1.51)	0.972
SIB (ref. No)															
Yes	0.92	(0.64–1.30)	0.639	0.92	(0.70–1.20)	0.527	0.94	(0.65–1.33)	0.713	0.93	(0.69–1.24)	0.616	0.89	(0.69–1.13)	0.334

Legend: OTT = overall treatment time; SIB = simultaneous integrated boost; HR PTV = high-risk planning target volume; LR PTV = low-risk planning target volume. HR = hazard risk; 95% CI = 95% confidence interval. In bold, statistically significant values (*p* < 0.05).

## Data Availability

Not applicable.
